# Cysteine Residues in the Major Capsid Protein, Vp1, of the JC Virus Are Important for Protein Stability and Oligomer Formation

**DOI:** 10.1371/journal.pone.0076668

**Published:** 2013-10-09

**Authors:** Shintaro Kobayashi, Tadaki Suzuki, Manabu Igarashi, Yasuko Orba, Noriko Ohtake, Keita Nagakawa, Kenichi Niikura, Takashi Kimura, Harumi Kasamatsu, Hirofumi Sawa

**Affiliations:** 1 Division of Molecular Pathobiology, Hokkaido University Research Center for Zoonosis Control, Kita-ku, Sapporo, Japan; 2 Global COE Program for Zoonosis Control, Hokkaido University, Sapporo, Japan; 3 Department of Pathology, National Institute of Infectious Diseases, Tokyo, Japan; 4 Division of Bioinfomatics, Hokkaido University Research Center for Zoonosis Control, Sapporo, Japan; 5 Graduate School of Chemical Science and Engineering, Hokkaido University, Sapporo, Japan; 6 Research Institute for Electronic Science (RIES), Hokkaido University, Sapporo, Japan; 7 Department of Molecular, Cell and Developmental Biology and Molecular Biology Institute, University of California Los Angeles, Los Angeles, California, United States of America; Centro de Biología Molecular Severo Ochoa (CSIC-UAM), Spain

## Abstract

The capsid of the human polyomavirus JC virus (JCV) consists of 72 pentameric capsomeres of a major structural protein, Vp1. The cysteine residues of the related Vp1 of SV40 are known to contribute to Vp1 folding, pentamer formation, pentamer-pentamer contacts, and capsid stabilization. In light of the presence of a slight structural difference between JCV Vp1 and SV40 counterpart, the way the former folds could be either different from or similar to the latter. We found a difference: an important contribution of Vp1 cysteines to the formation of infectious virions, unique in JCV and absent in SV40. Having introduced amino acid substitution at each of six cysteines (C42, C80, C97, C200, C247, and C260) in JCV Vp1, we found that, when expressed in HeLa cells, the Vp1 level was decreased in C80A and C247A mutants, and remained normal in the other mutants. Additionally, the C80A and C247A Vp1-expressing cell extracts did not show the hemagglutination activity characteristic of JCV particles. The C80A and C247A mutant Vp1s were found to be less stable than the wild-type Vp1 in HeLa cells. When produced in a reconstituted *in vitro* protein translation system, these two mutant proteins were stable, suggesting that some cellular factors were responsible for their degradation. As determined by their sucrose gradient sedimentation profiles, *in vitro* translated C247A Vp1 formed pentamers, but *in vitro* translated C80A Vp1 was entirely monomeric. When individually incorporated into the JCV genome, the C80A and C247A mutants, but not the other Vp1 cysteine residues mutants, interfered with JCV infectivity. Furthermore, the C80A, but not the C247A, mutation prevented the nuclear localization of Vp1 in JCV genome transfected cells. These findings suggest that C80 of JCV Vp1 is required for Vp1 stability and pentamer formation, and C247 is involved in capsid assembly in the nucleus.

## Introduction

The human pathogenic JC virus (JCV) is the causative agent of progressive multifocal leukoencephalopathy (PML), a fatal demyelinating disease of the central nervous system. It belongs to the polyomavirus family of nonenveloped, double-stranded DNA viruses, which also includes SV40, the BK virus (BKV), and murine polyomavirus (MPyV). The genomic DNA of polyomaviruses is housed in a virion structure, which consists of a capsid made from 72 pentamers of the major structural protein, Vp1. The 2 minor structural proteins, Vp2 and Vp3 (Vp2/3 for short), reside in the virion core with the viral DNA. Virion formation in the nucleus of the infected cell depends on the formation of Vp1 pentamers in the cytoplasm, followed by their transport to the nucleus where they interaction with Vp2/3 and with the viral genome [1]. JCV Vp1 is able to self-assemble into virus-like particles (VLPs) in the absence of Vp2/3 and viral genomic DNA when expressed in *Escherichia coli* (*E. coli*), yeast cells, or insect cells [2]–[4]. The structural properties of recombinant JCV VLPs are very similar to those of JCV virion particles [2]–[4].

The structures of the JCV capsid and Vp1 pentamer show that each Vp1 monomer has three modules: an N-terminal arm, an antiparallel β-barrel domain, and a long C-terminal extension [5], [6]. The JCV Vp1 pentamer consists of five Vp1 monomers which associate with neighboring monomers *via* their β-barrel domains [5]. The first 19 amino acids at the N-terminus and the last 31 amino acids at the C-terminus of JCV Vp1 are not essential for the formation of the pentamer [7]. In the SV40 capsid, Vp1 pentamer-pentamer contacts are formed *via* the long C-terminal arms extending from each pentamer into adjacent pentamers [8], [9], and these contacts occur between the G2H loop of each adjacent pentamer and the C-helix of each invading C-terminal arm [8], [9].

Cysteines residues in SV40 Vp1 (C9, C49, C87, C104, C207, C254 and C267) function at two distinct stages in the formation of SV40 capsid. The crystal structure of SV40 shows that there are no disulfide bonds in a pentamer or a monomer [8], [9]. However, transient disulfide bonds are formed during the SV40 Vp1 folding and pentamer formation [10]. Two sets of cysteine pairs found in C49A–C87A pair mutant andC87A–C254A pair mutant eliminate SV40 viability [11], while individual single mutations of seven SV40 cysteines largely preserve viral viability [12]. Furthermore, C49A–C87A pair mutant disrupts the formation of disulfide-linked SV40 Vp1 oligomers [13]. In the nuclear stage of SV40 virion assembly, mutation of C254, which exists at a junction between three pentamers and the twin calcium ions, interferes with pentamer-pentamer contacts [12]. Finally, structural analysis also indicates that C104–C104 disulfide bonds are observed between SV40 Vp1 pentamers [9] and that they stabilize the capsid structure [14].

**Figure 1:**
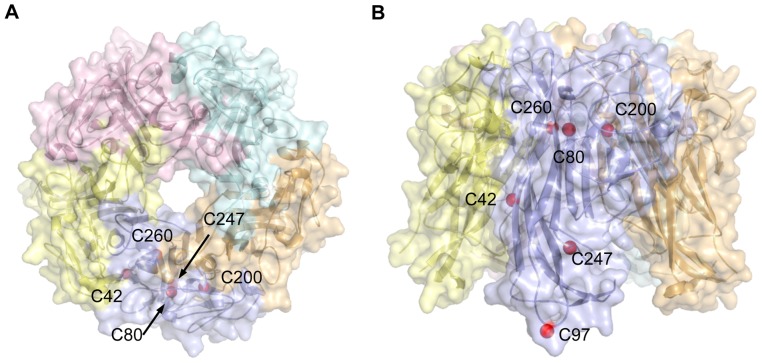
Cysteine residues in JCV Vp1. Three-dimensional model of the JCV Vp1 pentamer was constructed using data from an X-ray crystal structure of JCV Vp1 (PDB code: 3NXG) using the Discovery Studio 3.0 software package (Accelrys, San Diego, CA). Structure of the JCV Vp1 pentamer viewed on its top (A) and side (B) are shown as transparent surface and ribbon representation. Red spheres indicate the positions of cysteine residues on Vp1.

The JCV Vp1 shares about 75% amino acid sequence identity with the SV40 Vp1. A difference between their Vp1 pentamer structures suggests that mechanisms of the JCV capsid formation may differ from those of SV40. Of six cysteine residues at positions 42, 80, 97, 200, 247, and 260 in JCV Vp1, C80, C200, C247, and C260 are buried in the hydrophobic core of Vp1 (Fig. 1A and B), the distance between any two cysteine sulfur atoms on a pentamer being at least 10 Å [5]. How cysteine residues of JCV Vp1 functions in the formation of pentamer and capsid is currently unknown.

In the present study, we investigated the requirement of the JCV Vp1 cysteine residues for Vp1 folding and for JCV capsid formation by using a mutagenesis approach. We found that C80 is important for the conformational stabilization and pentamer formation of JCV Vp1. We also uncovered a possible importance of C247 in virion formation in the nucleus.

## Experimental Procedures

### Cells

Human cervical carcinoma (HeLa) cells were obtained from the Health Science Research Resource Bank (Osaka, Japan). *Atg5^+/+^* and *Atg5^−/−^* mouse embryonic fibroblasts (MEFs) [15] were obtained from the RIKEN BRC cell bank (Tsukuba, Japan). SV40-transformed human glial SVG-A cells were kindly provided by Dr. W. J. Atwood [16]. The cells were maintained under 5% CO_2_ at 37°C in DMEM supplemented with 10% heat-inactivated fetal bovine serum, penicillin, streptomycin, and 2 mM L-glutamine (Sigma, St. Louis, MO).

### Plasmids

For the expression of JCV Vp1 in HeLa cells, the plasmid pCXSN-Vp1 was constructed by subcloning the PCR-amplified JCV Vp1 coding sequence from pBR-Mad1 [17], which encodes the JCV genome, into pCXSN, which was created by removing the myc-tag from pCMV-myc (Clontech, Mountain View, CA) and adding Xho I, Sal I and Not I recognition sites [18]. For the synthesis of JCV Vp1 in a cell-free expression system, pURE2-Vp1 (Post Genome Institute, Tokyo, Japan) was constructed by subcloning the same JCV Vp1-encoding PCR amplicon into pURE2. pUC19-Mad1-SVEΔ, which contains the JCV Mad1-SVEΔ genome, a subclone of the Mad1-SVEΔ hybrid strain of JCV described previously [19], was kindly provided by Dr. W. J. Atwood [20], [21]. Cysteine mutants of JCV Vp1 were synthesized by using an inverse PCR method with pCXSN-Vp1, pURE2-Vp1, and pUC19-Mad1SVEΔ as templates, which allowed the cysteine residues at positions 42, 80, 97, 200, 247, and 260 to be individually replaced. Successful mutagenesis was confirmed by sequencing.

### Transfection and Immunoblotting

Plasmids were transfected into HeLa cells, *Atg5^+/+^* and *Atg5^−/−^* MEF cells, and SVG-A cells using the FuGENE HD reagent (Promega, Madison, WI) according to the manufacturer's instructions. For immunoblotting, cells were harvested at the indicated time points after transfection, lysed in TNE buffer (10 mM Tris-HCl [pH 7.5], 150 mM NaCl, 5 mM EDTA, 10% glycerol, 1% Triton X-100 and 0.5 mM PMSF), and supplemented with Complete Protease Inhibitor Cocktail (Roche Diagnostics, Hague Road, IN). The cell lysates were fractionated by SDS-PAGE, and the separated proteins were transferred to a PVDF filter (Millipore, Bedford, MA). The filter was incubated with a rabbit anti-JCV Vp1 polyclonal antibody, which was produced as described previously [22], and a mouse anti-actin monoclonal antibody, which was purchased from Millipore (Billerica, MA). The immune complexes were then detected with horseradish peroxidase-conjugated secondary antibodies (Biosource International, Camarillo, CA) and Immobilon Western HRP Substrate (Millipore). The chemiluminescence signals were visualized with the LAS-1000 Plus system (Fujifilm, Tokyo, Japan).

### Hemagglutination (HA) Assays

JCV VLP formation properties were examined by performing HA assays with human type-O erythrocytes as described previously [22]. Human type-O erythrocytes were provided by the Hokkaido Red Cross Blood Center (Sapporo, Japan). Briefly, cells expressing JCV Vp1s were extracted via exposure to three freeze-thaw cycles in 50 µl of Tris-HCl (pH 7.5) containing 0.2% BSA. The extract was incubated for 15 h at 37°C with 0.05 U/ml of neuraminidase type V (Sigma). After inactivation of neuraminidase for 30 min at 56°C, the extract was centrifuged at 600× *g* for 10 min. The resulting supernatant was then analyzed using to the HA assay. Two-fold serial dilutions of the cell extracts were made in PBS (pH 7.15) containing 0.2% BSA in 96-well, V-bottomed microplates. An equal volume of 0.5% red blood cells in PBS (pH 7.15) was added to each well and incubated at 4°C for 3 h.

### Metabolic Radiolabeling and Pulse Chase Analysis

HeLa cells expressing the wild type (WT) or a mutant JCV Vp1s were washed and incubated in DMEM without methionine and cysteine (Life Technologies, Medical Center Drive, MD) for 60 min at 37°C. After this starvation period, the medium was removed, and the cells were incubated in 0.5 ml of DMEM without methionine and cysteine and with added [^35^S] methionine and [^35^S] cysteine (0.3 mCi per 3.5 cm well; Perkin Elmer, Wellesley, MA) at 37°C for 5 min. After the labeling, the medium was removed, and the cells were washed with DMEM and either harvested immediately (5 min pulse label) or further incubated in DMEM for 12 or 24 h (chase). The cell of each well was harvested in 350 µl of TNE buffer. The lysates were immunoprecipitated with 3 µg of rabbit anti-JCV Vp1 antibody. Immunoprecipitation was performed by incubating cell lysates at room temperature for 15 min with 30 µl of antibody-coupled Dynabeads Protein A (Life Technologies). The precipitated protein complexes were separated by SDS-PAGE, and the gel was exposed and analyzed by using a BAS 2500 bio-image analyzer (Fujifilm).

### Vp1 Crystal Structure Comparison

The crystal structure of JCV Vp1 (PDB code: 3NXG) was superimposed on the structure of MPyV Vp1 (PDB code: 1VPN) or SV40 Vp1 (PDB code: 1SVA). Structural comparison images were prepared using PyMOL (http://www.pymol.org/).

### *In Vitro* Transcription-Coupled Translation

*In vitro* transcription and translation were carried out using the PURESYSTEM S-S kit (Wako, Osaka, Japan) [23], [24]. In this cell-free expression system, all factors for transcription and translation were tagged with hexahistidine, including 3 initiation factors (IF1, IF2 and IF3), 3 elongation factors (EF-G, EF-Tu and EF-Ts), 3 release factors (RF1, RF2 and RF3), ribosome recycling factor, 20 aminoacyl-tRNA synthetases, methionyl-tRNA formyltransferase, and T7 RNA polymerase. The reaction mixtures also contained *E. coli* 70S ribosomes, amino acids, NTPs, *E. coli* tRNAs, and an energy recycling system, resulting in target protein synthesis immediately after the addition of template DNA. PURESYSTEM S-S is specially designed to enable disulfide bond formation in the synthesized protein. For the cell-free synthesis of each of the JCV Vp1, the corresponding pURE2-Vp1 template plasmid, which contains a T7 promoter, a ribosomal binding site, and a terminator sequence, was mixed with the PURESYSTEM S-S mixture and incubated at 37°C for 1 h. The whole reaction mixture was then subjected to immunoblotting and sedimentation analyses.

### Sedimentation Analysis

For nondenaturing sedimentation, protein samples (100 µl) were centrifuged through 4.2 ml of a 5–20% continuous sucrose gradient with 0.3 ml of a 50% sucrose cushion. The sucrose solutions were prepared in 50 mM HEPES-NaOH (pH 7.5), 140 mM NaCl. The gradients were centrifuged at 40,000 rpm (194,000× *g*) (SW55 rotor, Beckman Coulter, Brea, CA) at 20°C for 13 h and then fractionated from the top into 22 fractions of 220 μl each. Fractions 1 to 18 were analyzed by SDS-PAGE and immunoblotting using a rabbit anti-JCV Vp1 antibody.

Denaturing sedimentation was performed in a similar way, except that the sucrose solutions also contained 0.1% SDS and that the protein samples were incubated for 30 min at 50°C in 50 mM HEPES-NaOH (pH 6.8), 0.5% SDS, 1% NP-40 and 2 mM N-ethylmaleimide (NEM), a sulfhydryl alkylation reagent, before being loaded onto the gradients.

### Expression of VLPs and Purification of Pentamers

JCV VLPs were prepared as previously described [22]. Briefly, the pET15b plasmid (Novagen, Madison, WI) encoding WT or mutant JCV Vp1 was transformed into BL21 (DE3) pLysS competent cells (Stratagene, La Jolla, CA). After overnight incubation at 37°C in Terrific Broth, Vp1 expression was induced using 1.0 mM isopropyl-β-D-thiogalactopyranoside (IPTG) for 4 h at 30°C, and the mixture was collected by centrifugation at 4,000× *g* for 15 min. The pellet was resuspended in reassociation buffer (20 mM Tris-HCl [pH 7.4], 150 mM NaCl, and 1 mM CaCl_2_) containing 1 mg/ml of lysozyme and kept on ice for 30 min before the addition of 1% sodium deoxycholate. After incubation for 10 min on ice, the sample was treated with DNase I (100 U/ml) for 30 min at 30°C and lysed by five sonication cycles (30 sec each). After lysate centrifugation at 10,000× *g* for 20 min at 4°C, the supernatant was centrifuged at 25,000 rpm (112,500× *g*) for 3 h at 4°C (SW28 rotor, Beckman Coulter). The white layer in the tube was lysed in a reassociation buffer containing CsCl at a final concentration of 1.29 g/ml and then centrifuged at 32,000 rpm (175,300× *g*) for 16 h at 4°C (SW41 rotor, Beckman Coulter). The resultant VLP fractions were dialyzed in reassociation buffer at 4°C overnight.

For preparation of the Vp1 pentamer, purified VLPs were adjusted to 25 mM EGTA and 30 mM DTT, incubated for 1 h at 37°C, and separated on a Superdex 200 gel filtration chromatography column (GE Healthcare, Uppsala, Sweden) in 20 mM Tris-HCl (pH 8.0), 150 mM NaCl, 5 mM EGTA, and 5 mM DTT at 4°C. The peak fractions that were predicted to comprise pentamers (having a molecular weight of approximately 220 kDa) were collected and stored at −80°C.

### Electron Microscopy

Before observation, samples (2 µl) were dropped onto collodion-coated transmission electron microscope grids (Nisshin EM, Tokyo, Japan) and then negatively stained with 2% phosphotungstic acid. VLP and pentamer morphologies were confirmed by scanning transmission electron microscopy (STEM) (HD-2000, Hitachi, Tokyo, Japan).

### JCV Infectivity Assays

Mutations at the cysteine residues in the Vp1 coding segment of pUC19-Mad1SVEΔ were introduced by an inverse PCR method. Mutant and WT JCV DNAs were isolated from the pUC19-Mad1SVEΔ template by digestion with the endonuclease Bam HI, and equal amounts of the resulting viral DNAs were transfected into permissive SVG-A cells. Twelve days after transfection, the cells were analyzed for the expression of agnoprotein by indirect immunofluorescence using a rabbit anti-agnoprotein polyclonal antibody, which was produced as described previously [25]. Results were confirmed by performing at least three independent experiments.

### Indirect Immunofluorescence Analysis

SVG-A cells seeded onto glass-bottomed dishes (Iwaki, Tokyo, Japan) were transfected with WT or mutant JCV genomes. Three days after transfection, the cells were fixed for 3 min in 100% methanol at room temperature, blocked with 1% BSA to prevent nonspecific antibody binding, and incubated overnight with mouse anti-Vp1 (Abcam, Cambridge, MA) and rabbit anti-agnoprotein antibodies at 4°C. Immune complexes were visualized by incubation with Alexa Fluor 488-conjugated goat antibody to mouse IgG or with Alexa Fluor 594-congugated goat antibody to rabbit IgG (Life Technologies) for 1 h at room temperature. Cell nuclei were counterstained with DAPI (Life Technologies). The cells were then observed using a confocal laser-scanning microscope (Olympus, Tokyo, Japan).

### Statistical Analysis

All data are expressed as means ± SD. Student's *t*-tests were performed to determine the significance of differences between samples. Values of *p*<0.05 were regarded as significant.

## Results

### Formation of VLP in HeLa Cells Is Interrupted by Mutation of JCV Vp1 C80 or C247 into Alanine

**Figure 2:**
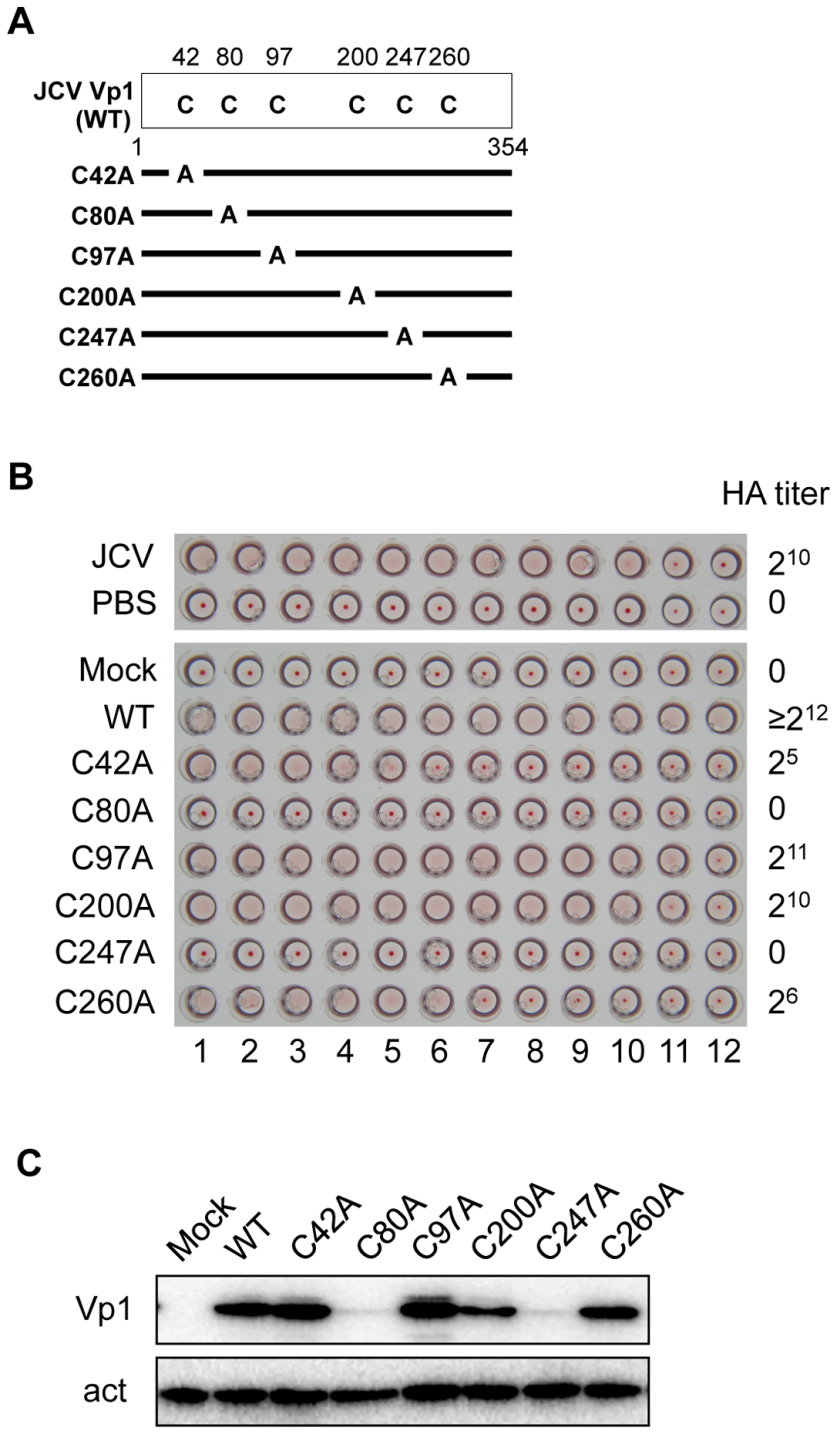
HA titers and Vp1 expression levels of JCV Vp1 cysteine point mutants in mammalian cells. (A) Schematic diagram of cysteine point mutant Vp1s. Each of the six cysteine residues in the 354-amino-acid JCV Vp1 is marked with a “C” for the WT Vp1 (WT), and the cysteine residue mutated into alanine is designated as “A” for each point mutant Vp1. (B) HA titers of extracts from Vp1-expressing cells. HA titers were determined from the last dilution showing hemagglutination for two-fold serially diluted extracts prepared from HeLa cells transfected with the expression plasmids for WT or individual point mutant Vp1s. The corresponding dilutions of extract from JCV-infected cells served as a positive control, whereas those of PBS or of HeLa extract from transfection with the empty plasmid (Mock) were used as negative controls. (C) Levels of Vp1 expression. Quantitation of the mutant Vp1s and actin in HeLa cells transfected with the Vp1 expression plasmids or with an empty vector (Mock) were analyzed by SDS-PAGE and immunoblotting for Vp1 (Vp1) and for actin (act).

JCV virions possess hemagglutination (HA) activity toward O-type human red blood cells [26], and the presence of HA activity indicates successful formation of VLPs from JCV Vp1 expressed in *E. coli* [4]. We first examined whether JCV Vp1 alone can form particles in HeLa cells and whether any of the Vp1 cysteine residues play a role in this process. Six individual cysteine-to-alanine substitution mutations (C42A, C80A, C97A, C200A, C247A, and C260A) were introduced into a mammalian expression plasmid that encodes WT JCV Vp1 (Fig. 2A, WT), and the resulting plasmids were transfected into HeLa cells. As a positive control, we first examined whether extracts from JCV-infected cells had HA activity, and they did, as expected (Fig. 2B, JCV). We next tested the HA activities of extracts from cells expressing WT Vp1 or the six individual mutant Vp1s. HA activity was present in the WT Vp1 extract (≥2^12^ HA titer in 25 μl; Fig. 2B, WT) but not in the extract of mock- treated cells (Mock). Among the six mutants, three categories of HA activities could be seen. First, the extracts from cells expressing C97A and C200A Vp1s had two- to four-fold lower HA titers than the WT sample (Fig. 2B, C97A and C200A). In the second category, the extracts of C42A and C260A Vp1s had about 100-fold lower HA titers than the WT sample (Fig. 2B, C42A and C260A). Third, the extracts of C80A and C247A Vp1s transfected cells did not show any signs of HA (Fig. 2B, C80A and C247A). These results show that the extracts from HeLa cells expressing JCV Vp1 can support the formation of VLPs that exhibit HA activity, a characteristic of JCV virions. VLP formation was abrogated when C80 or C247 was changed to alanine, consistent with the idea that these two cysteines have significant roles in VLP formation.

A lack of HA activity in a mutant sample could result from an extremely low level of mutant protein in the sample. When we examined the effect of cysteine mutations on the steady-state level of Vp1 in the HeLa expression system, we observed that the levels of both C80A and C247A mutant Vp1s were markedly reduced, whereas the levels of C42A, C97A, C200A, and C260A mutant Vp1s were similar to that of WT Vp1 (Fig. 2C).

### C80A and C247A Mutant Vp1s Are Less Stable than WT Vp1

**Figure 3:**
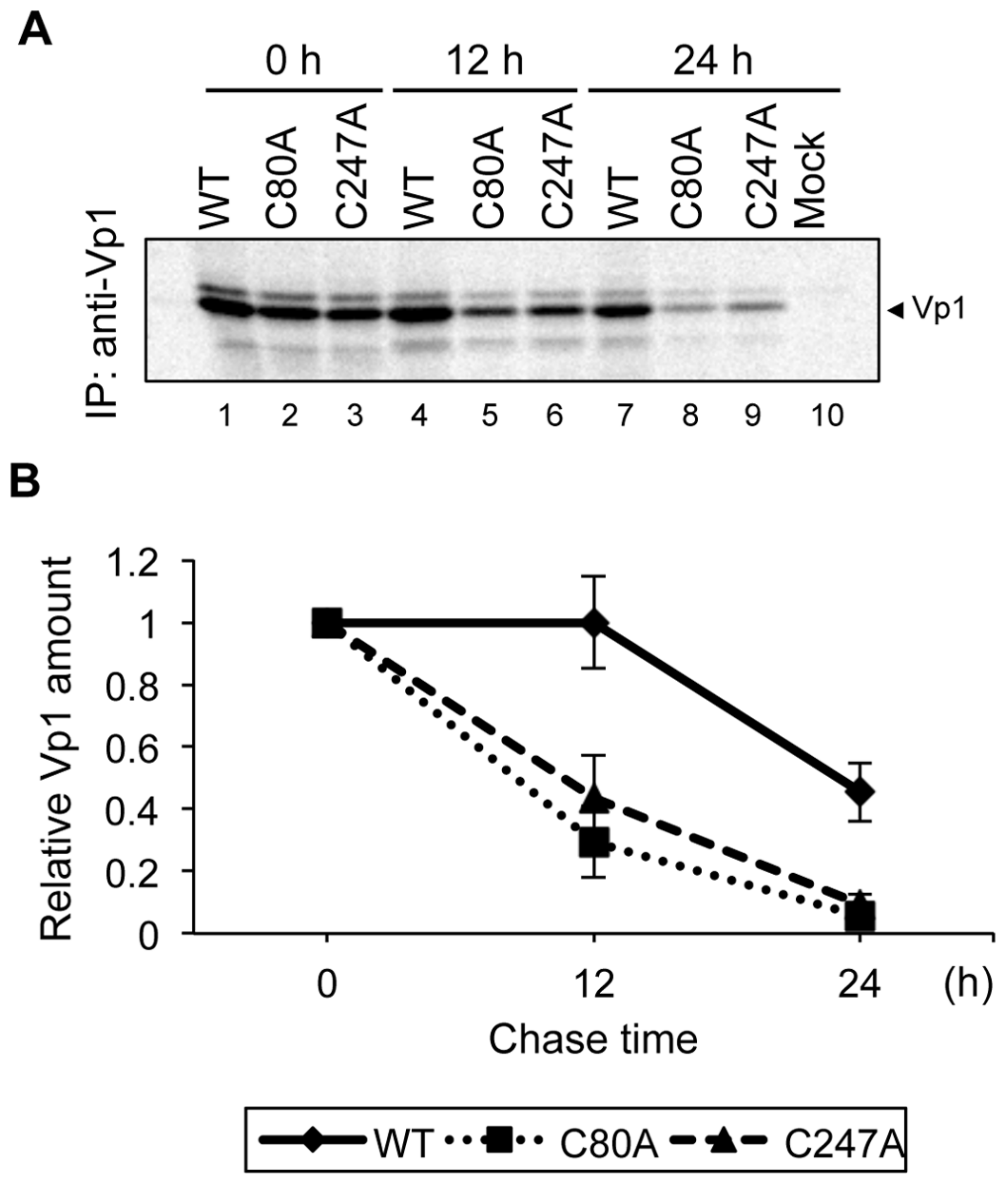
Stabilities of WT Vp1 and C80A and C247A mutant Vp1s expressed in HeLa cells. (A) HeLa cells transfected with an empty plasmid (Mock) or plasmid encoding WT, C80A, or C247A Vp1 were pulse-labeled for 5 min and then either harvested immediately (lanes 1 to 3) or chased for 12 h (lanes 4 to 6) or 24 h (lanes 7 to 10). The anti-Vp1 immunocomplexes (IP) prepared from the lysates were analyzed by SDS-PAGE and autoradiography. (B) The intensities of the bands in *A* were quantified with an image analyzer and plotted as relative values compared to those obtained at 0 h of chase. Data are the means ± S.D. of values from three independent experiments. The solid line, dotted line, and broken line represent WT, C80A, and C247A samples, respectively.

We next investigated why C80A and C247A mutant Vp1s failed to accumulate in transfected HeLa cells by performing pulse-chase experiments. Cells transfected with WT, C80A, and C247A Vp1 expression plasmids were radiolabeled for 5 min and either harvested immediately or chased in normal growth medium for 12 h or 24 h before harvesting. Cell lysates were prepared and subjected to anti-JCV Vp1 immunoprecipitation, and the labeled immunocomplexes were visualized by fluorography. The amounts of the two newly synthesized mutant Vp1s immediately after pulse labeling were similar to that of WT Vp1 (Fig. 3A, 0 h, lanes 1–3). The intensity of the WT Vp1 band remained the same for 12 h afterward, but was reduced to half by 24 h of chase (Fig. 3A. lanes 1, 4, and 7). In contrast, the label intensities of C80A and C247A mutant Vp1s became progressively lower after 12 h and 24 h of chase (Fig. 3A, lanes 5, 6, 8 and 9). That both mutant Vp1s were unstable relative to WT Vp1 is further shown in Fig. 3B, in which the amount of each Vp1 at each time point was quantitated by densitometry and normalized to the amount of Vp1 detected at 0 h. These results show that C80A and C247A mutant Vp1s were synthesized as efficiently as WT Vp1, but they were less stable and apparently were degraded during the chase-periods.

### Structural Perturbation at C80 and C247 May Affect JCV Vp1's Steady State Level in HeLa Cells

**Figure 4:**
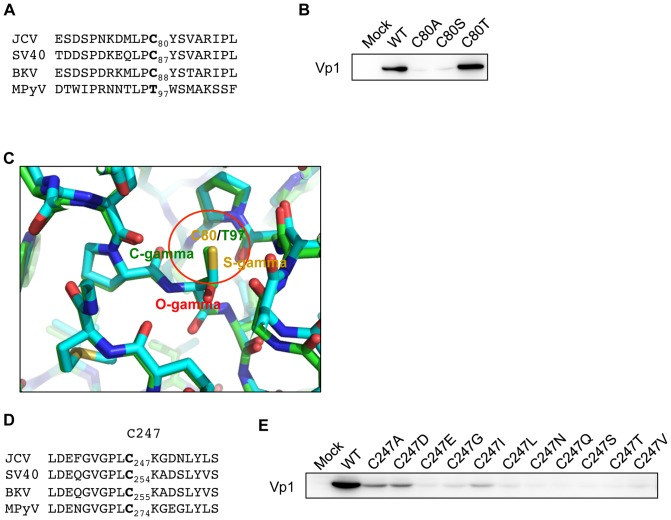
Structural environments of JCV Vp1 C80 and C247 in relation to Vp1 stability. (A) Alignment of amino acid sequences of JCV, SV40, BKV, and MPyV Vp1s in the vicinity of C80. Amino acids homologous to C80 are in boldface. (B) C80T mutant Vp1, but not C80A or C80S Vp1, is stable. Lysates of HeLa cells were analyzed by SDS-PAGE and immunoblotting for Vp1. These cells were prepared 48 h after transfection with the expression plasmid encoding eithe WT Vp1 or each C80 mutant Vp1 or after transfection with empty expression plasmid (Mock). (C) Comparison of the structures of JCV and MPyV Vp1s surrounding C80. The JCV structure is superimposed on the MPyV structure. The sulfur atom of JCV's C80 and the methyl group of MPyV's T97 are circled in red. (D) Alignment of amino acid sequences of JCV, SV40, BKV, and MPyV Vp1s in the vicinity of C247. Amino acids homologous to C247 are in boldface. (E) All C247 substitution mutant Vp1s expressed in HeLa cells are unstable. Lysates of HeLa cells transfected with the expression plasmid for WT Vp1 or for each C247 mutant Vp1 or after transfection with empty expression plasmid (Mock) were analyzed for Vp1 by SDS-PAGE and immunoblotting for Vp1.

Fig. 4A shows the amino acid sequence context of C80 in JCV Vp1, in alignment with those of the structurally equivalent residues in other polyomavirus Vp1s. A cysteine is present in SV40 and BKV at the position of JCV C80, whereas a threonine is present in mouse polyomavirus (MPyV). We thus substituted the C80 of JCV Vp1 with serine (C80S), a conservative side-chain change from cysteine, and with threonine (C80T), the amino acid present at the equivalent MPyV Vp1 position. Both C80S and C80A Vp1s showed reduced steady-state levels, whereas the steady-state level of C80T Vp1 was equivalent to that of WT Vp1 (Fig. 4B). We overlaid the local structure of JCV Vp1 on the corresponding structure of MPyV Vp1 (Fig. 4C). JCV Vp1 [5] and MPyV Vp1 [27] form superimposable secondary and tertiary structures. The Vp1 region that includes the C80 of JCV Vp1 shows C80 lying on the BC loop connecting two antiparallel β-sheet strands; this loop is a part of Vp1's hydrophobic core [5]. The sulfur atom of C80 does not make any specific interactions in JCV Vp1 and fills a hydrophobic space (Fig. 4C, region encircled in red). The C-γ methyl group of the threonine in MPyV Vp1 occupies the space of the cysteine sulfur. A cysteine-to-alanine or cysteine-to-serine mutation at position 80 would leave a hole in the hydrophobic core of JCV Vp1, which is probably energetically unfavorable. These results suggest that the structural integrity of the Vp1 region that includes C80 is important for maintaining the protein in a stable state.

The sequences of amino acids flanking the C247 of JCV Vp1 are well conserved among the Vp1s of polyomaviruses [12]. The amino acid contexts surrounding JCV Vp1 C247 and the analogous cysteines of SV40, BKV, and MPyV are shown in Fig. 4D. To examine whether the amino acid side chain at position 247 of JCV Vp1 influences the steady-state level of Vp1, we substituted C247 with each of the ten amino acids (C247D, C247E, C247G, C247I, C247L, C247N, C247Q, C247S, C247T, and C247V). The steady-state levels of all eleven C247 substitution mutant Vp1s, including that of the C247A mutant, were low compared with that of the WT (Fig. 4E). The fact that any substitution at C247 led to an unstable mutant protein suggests that this cysteine residue must serve as a key determinant for Vp1 assembly.

### C80A and C247A Mutant Vp1s Synthesized in Vitro Are Stable

The results presented in Fig. 3 suggest that C80A and C247A mutant Vp1s, with low steady-state levels, are possibly recognized by degradation factors in host cells. Two major protein degradation systems known to operate in eukaryotic cells are (i) the ubiquitin-proteasome system and (ii) autophagy [28]. Either of these degradation systems, or working in concert, could diminish the steady state levels of the mutant Vp1s. We examined the intracellular stability of the mutant Vp1s in the presence of a proteasome inhibitor and in cells incapable of autophagy [15]. The results were inconclusive; the level of C80A and C247A Vp1s remained low whether expressed in HeLa cells treated with the proteasome inhibitor MG-132 or expressed in *Atg5* KO mouse embryonic fibroblasts (MEFs), which lacks the capacity for autophagy (data not shown). These exploratory results imply the presence of unique cellular components other than the known proteasome or autophagy degradation systems that recognize the structure-based abnormalities of the mutant Vp1s and promote their degradation, hence rendering the proteins unstable.

**Figure 5:**
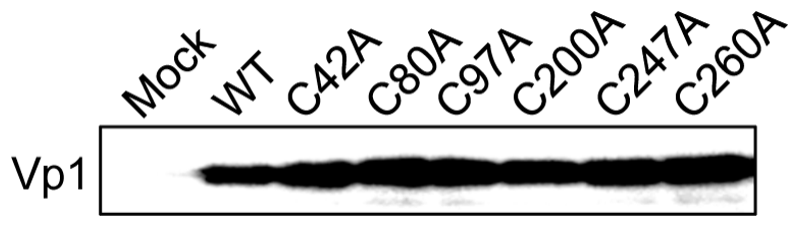
Cysteine point-mutant Vp1s synthesized *in vitro* are stable. The empty pURE2 plasmid (Mock) or pURE2-Vp1 plasmids encoding either WT Vp1 or cysteine point mutant Vp1s were subjected to cell-free transcription-coupled translation, and the translation products were examined for Vp1 by SDS-PAGE and immunoblotting.

We therefore tested whether C80A and C247A mutant Vp1s could be stable when synthesized *in vitro*. We chose an *in vitro* protein synthesis system consisting of isolated components for transcription and translation machineries and containing oxidizing agents and disulfide bond isomerase to enable disulfide bond formation in the protein products. This system is also free of components required for protein degradation or modification [23], [24]. The transcription of input Vp1 expression plasmids, coupled with translation, supported the synthesis of WT and mutant Vp1s. We detected steady-state levels of C80A and C247A Vp1s made during one hour's reaction time in this cell-free system were the same as those of WT and other cysteine mutant Vp1s, C42A, C97A, C200A, and C260A (Fig. 5). All mutant protein products remained stable, in contrast to the same mutant Vp1s expressed in mammalian cells (compare Figs. 2C with Fig 5). These results suggest that HeLa cells contain cellular factors that recognize structure-based abnormalities and that such proteins recognized C80A and C247A mutant Vp1s as unfit and targeted them for degradation.

### Single Cysteine-to-Alanine Substitution Mutant Vp1s Made In Vitro Exhibit Subtly Different Biochemical Properties

Since the stability of C80A and C247A mutant Vp1s was compromised in HeLa cells, the proteins' oligomerization properties could not be tested in that system. We examined whether the cysteine-to-alanine substitution mutant Vp1s, in particular C80A and C247A Vp1s, could form oligomers when synthesized *in vitro*. The products were sedimented through sucrose gradients, and the presence of mutant Vp1s in the resulting fractions was examined to assess the extent of Vp1 oligomerization.

**Figure 6:**
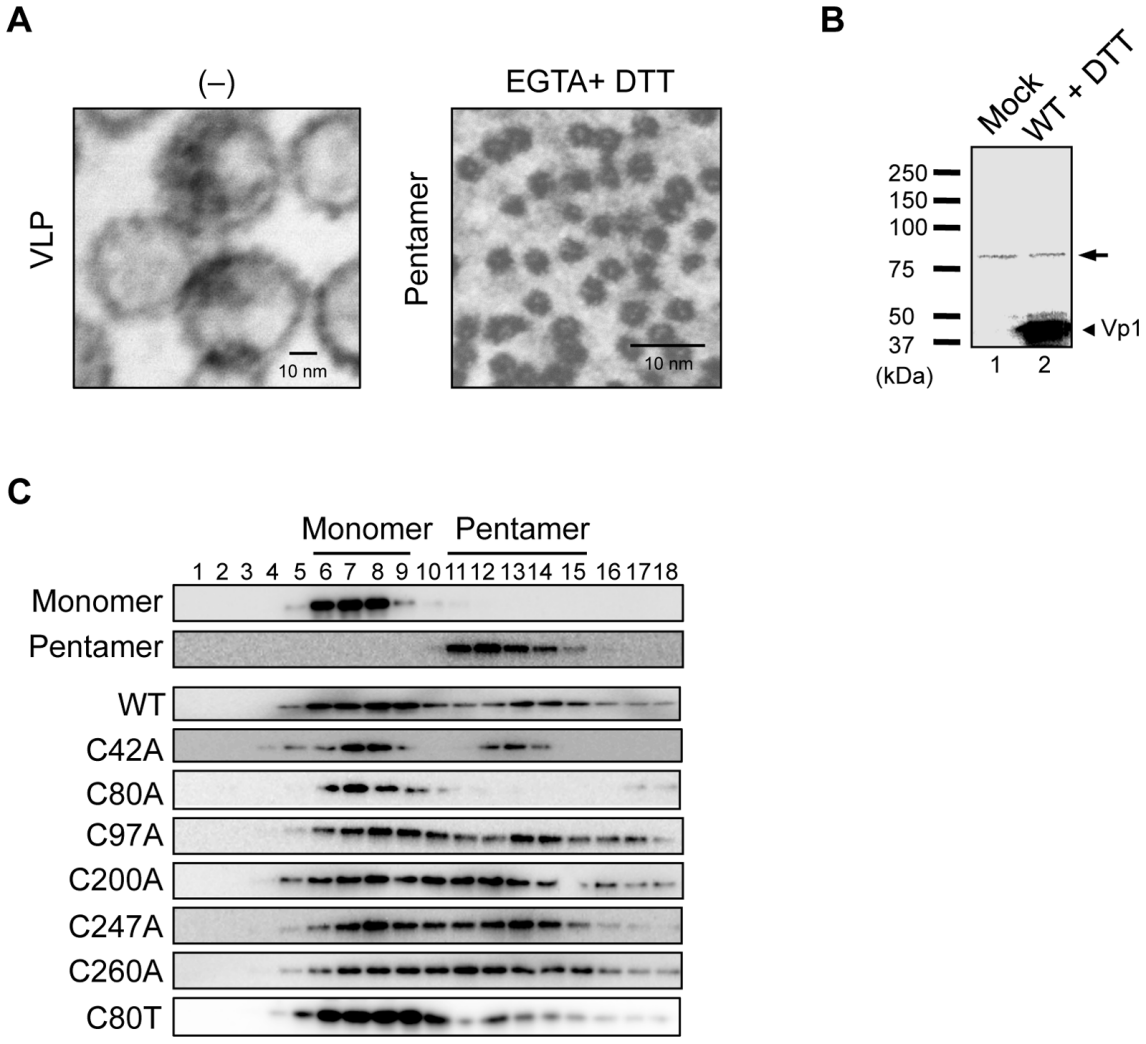
Oligomerization analysis of *in vitro* synthesized WT and mutant Vp1s. (A) Electron micrographs of VLPs (VLP; scale bar, 10 nm) and pentamers (EGTA + DTT; scale bar, 10 nm) formed by WT JCV Vp1. (B) The mixtures resulting from the *in vitro* transcription-coupled-translation of the empty pURE2 plasmid (Mock) and of the Vp1-encoding pURE2-Vp1 (WT) were supplemented with DTT, boiled, separated by SDS-PAGE, and immunoblotted with a rabbit anti-Vp1 antibody. An arrowhead marks the position of monomeric Vp1. An arrow indicates nonspecific bands. (C) Sedimentation profiles of *in vitro* translated single cysteine substitution mutants. Monomeric Vp1s, pentameric Vp1s, and the *in vitro* translation products for WT Vp1 or cysteine point mutant Vp1s (C42A, C80A, C97A, C200A, C247A, C260A, or C80T) were separated by 5–20% sucrose gradient sedimentation under denaturing conditions, and the resulting fractions were examined for the presence of Vp1 by SDS-PAGE and immunoblotting.

We first determined which sedimentation fractions JCV Vp1 pentamera and monomers are located in. Vp1 pentamers were prepared by dissociating JCV VLPs with DTT and EGTA, as previously reported [14]. The appearances of the VLPs and of the pentamers following the dissociation treatment were distinct on electron micrographs (Fig. 6A). The observed spherical JCV VLPs and the chemically disrupted pentamers were similar in size to the reported SV40 Vp1 VLPs and pentamers, respectively [14]. The JCV Vp1 pentamers were sedimented through nondenaturing sucrose gradients and were found in fractions 11 through 15 of the gradient (Fig. 6C, Pentamer). To determine in which fractions the JCV Vp1 monomers would be present, WT Vp1 synthesized *in vitro* were denatured in the presence of SDS and DTT, confirmed of the correct size by SDS-PAGE and immunoblotting by anti-Vp1 antibody, and subjected to nondenaturing sucrose sedimentation. The presence of Vp1 in the resulting fractions was probed by immunoblotting. The relative mobility of the monomeric Vp1 in SDS-PAGE was approximately 44 kDa, consistent with the theoretical molecular weight (Fig. 6B). Such Vp1 monomers were found in fractions 6 through 9 of the gradient (Fig. 6C, Monomer).

WT and mutant Vp1s synthesized *in vitro* were similarly fractionated by sedimentation through denaturing gradients to disrupt noncovalently associated proteins, including those of the protein synthesis machinery. The distribution of WT and mutant Vp1s in the fractions was assessed by anti-Vp1 immunoblotting. For WT Vp1, intense Vp1 signals were detected in fractions 6 through 9 and 13 through 15, corresponding to the locations of the monomers and pentamers, respectively (Fig. 6C, WT), indicating pentamer formation by WT Vp1. Similarly for C42A, C97A, C200A, C247A, and C260A mutant Vp1s, Vp1 signals were found in both the monomer and pentamer regions of the denaturing gradients (Fig. 6C, C42A, C97A, C200A, C247A, and C260A). A different sedimentation pattern was observed for *in vitro* synthesized C80A Vp1. The C80A mutant Vp1s was found almost entirely in the fractions expected for monomers (Fig. 6C, C80A). The C80A mutant Vp1 appears to form a local structure that prevent in forming intermolecular interaction of the mutant monomers. This interpretation was verified: The C80T mutant Vp1 synthesized *in vitro* was detected in both the monomer and pentamer fractions (Fig. 6C, C80T). These results show that local structure of C80 of JCV Vp1 is important for the Vp1 pentamer formation. C247, on the other hand, is not essential for Vp1 pentamer formation.

### The Infectivities of C80A and C247A Mutant JCVs Are Decreased

**Figure 7:**
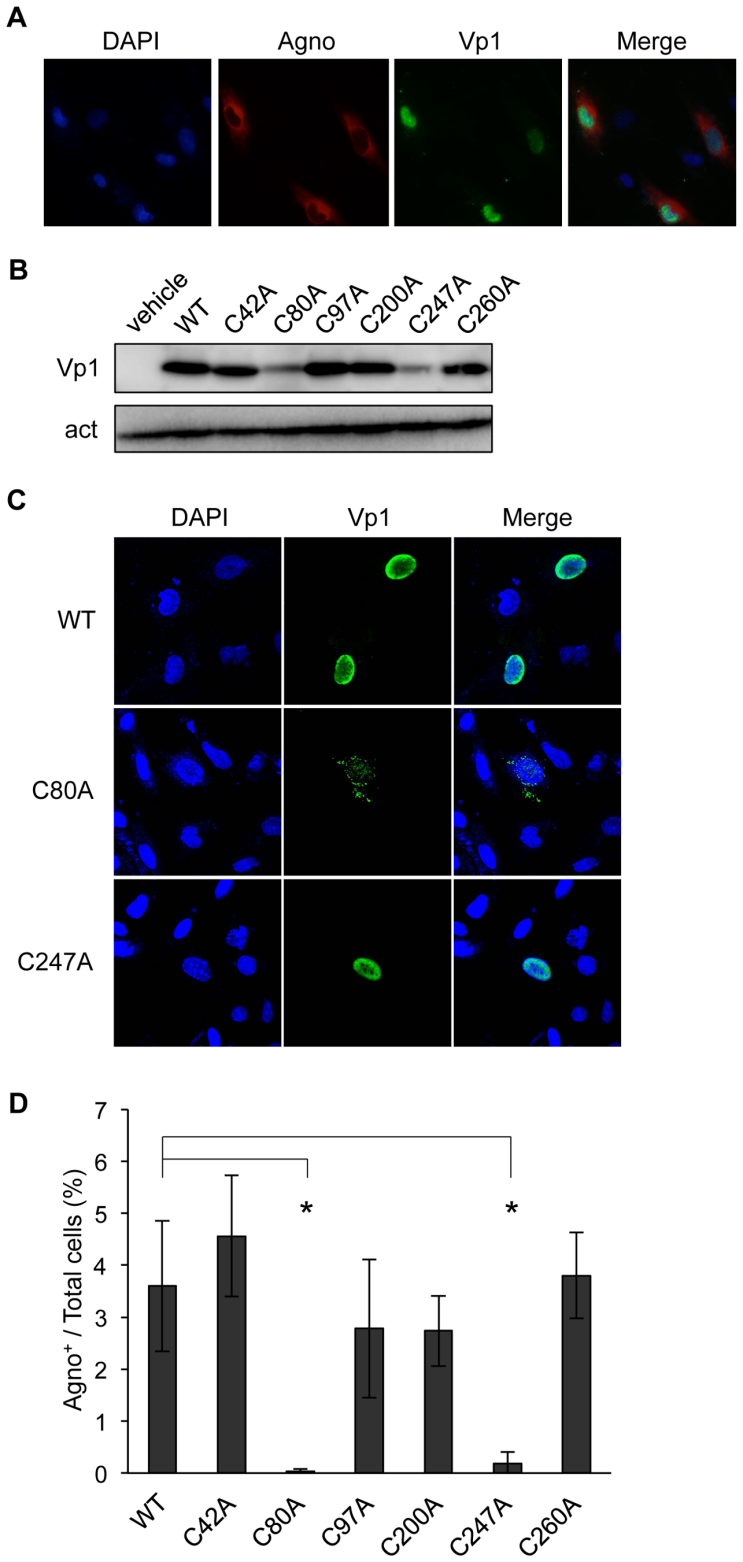
C80A and C247A mutations decrease infectivity. (A) Expression of JCV-encoded agnoprotein and Vp1 after JCV genome transfection. SVG-A cells were transfected with WT JCV genome for 3 days, fixed, and examined for the presence of agnoprotein and Vp1 by immunofluorescence analysis. Cell nuclei were counterstained with DAPI. Merged images of DAPI (Blue), Agno (Red), and Vp1 (Green) are also presented. (B) Levels of Vp1 expression. SVG-A cells were transfected with JCV genomes encoding either WT Vp1 or cysteine point mutant Vp1s or transfected with the transfection reagent alone (vehicle) for 3 days, and cell lysates were analyzed by SDS-PAGE and immunoblotting for Vp1 (Vp1) and for actin (act). (C) Subcellular localization of C80A and C247A Vp1s. SVG-A cells at 3 days posttransfection with the WT or either mutant JCV genome were processed for immunofluorescence analysis with a mouse anti-Vp1 antibody (*green*). Cell nuclei were counterstained with DAPI (*blue*). Merged images of DAPI and Vp1 are also presented. (D) Infectivity of WT and mutant JCV genomes. JCV genomes encoding either WT or individual cysteine mutant Vp1s were transfected into SVG-A cells, and the effectiveness of infection initiation was determined by immunofluorescence analysis for the presence of agnoprotein. For each genome, the average percentage of cells positive for agnoprotein is presented in the bar graph as the mean ± SD of three microscopic fields. The data represent the mean ± SD of three independent experiments. The significance of the changes was analyzed by Student's *t*-test (*p<0.05).

Finally, we investigated whether the JCV Vp1 cysteines play a role in JCV infection. Transfection of the WT JCV genome into JCV-permissive SVG-A cells leads to a JCV infection [20], [21]. We used this genome transfection system to assess the biological activity of the cysteine mutant JCVs and tested whether JCV viral genomes mutated at only one of the six cysteines differ in their infectivity. The Vp1 mutations described above (Fig. 2A) were introduced into the JCV genome, creating mutant JCV viral DNAs carrying WT large T, small t, agnoprotein, Vp2/3, and mutant Vp1s harboring the individual cysteine mutations (C42A, C80A, C97A, C200A, C247A, and C260A). The viral DNAs were transfected into SVG-A cells. At 3 days post-transfection, the JCV viral genome encoding agnoprotein and WT Vp1 were detected in the SVG-A cells (Fig. 7A). Since SVG-A cells constitutively express SV40 large T, we could not distinguish between the JCV large T derived from the transfected JCV genome and the SV40 large T.

For mutant genome transfections, the steady-state levels of C42A, C97A, C200A, and C260A mutant Vp1s were comparable to that of WT Vp1 (Fig. 7B). At 3 days post-transfection, the levels of C80A and C247A mutant Vp1s expressed were reduced to approximately half that of WT and other cysteine mutant Vp1s (Fig. 7B). This result corroborates our observations in Vp1 plasmid-transfected HeLa cells (Fig. 2B).

The presence of Vp1 in cells transfected with JCV viral genomes was examined by immunofluorescence analysis after 3 days of transfection. The Vp1s were mainly detected in the nuclei of cells transfected with WT, C42A, C97A, C200A, C247A, and C260A mutant viral genomes (Figs. 7C and 1S). Although we observed the C247A mutant Vp1 in the nucleus, the C80A Vp1 was mainly detected as speckles in the cytoplasm (Fig. 7C).

The infectivity of the mutant Vp1 JCVs was determined by adopting a JCV infectivity assay to detect for the agnoprotein immunostaining of the transfected SVG-A cells. The proportion of agnoprotein-positive cells 12 days after transfection with C42A, C97A, C200A, or C260A mutant genome was approximately equivalent to that following transfection with WT JCV genome. The proportion of agnoprotein-positive cells following transfection with C80A or C247A mutant genome was significantly reduced (Fig. 7D). These results show that C80 and C247 have important roles in JCV propagation and infection, consistent with the result that these cysteines have significant roles in VLP formation.

## Discussion

The formation of JCV, the infectious human pathogen that causes PML, requires the proper assembly of the viral capsid from the major structural protein Vp1. Cysteine residues of SV40 Vp1 contribute to Vp1 folding, oligomerization, capsid assembly, and capsid stabilization [10]–[12], [14]. Because JCV Vp1 is structurally different from SV40 Vp1 [5], it has been assumed that the cysteine residues in JCV Vp1 have a different role than those in SV40 Vp1. Our study revealed that the structural integrity of the Vp1 region, which includes C80, is important for the protein stability of Vp1 and Vp1 pentamer formation. We also found that C247 is important for capsid formation in the nuclear stage of virion assembly. These cysteine residues do not participate in the formation of disulfide bonds in Vp1 pentamer formation.

We have shown that JCV Vp1 contains all of the determinants for capsid assembly in HeLa cells. When expressed in HeLa cells, WT Vp1 and C42A, C97A, C200A, and C260A mutant Vp1s, but not C80A and C247A mutant Vp1s, had HA activity, just as intact JCV virions do, and hence can form VLPs. This result demonstrates that the Vp1 protein alone is sufficient to produce HA activity in mammalian cells as well as yeast cells [3]. Studying how the amino acids of JCV Vp1 help to promote capsid formation has been hampered by the slow growth of JCV in cultured cells, and mammalian expression systems may offer a new approach for investigating JCV capsid formation.

**Figure 8:**
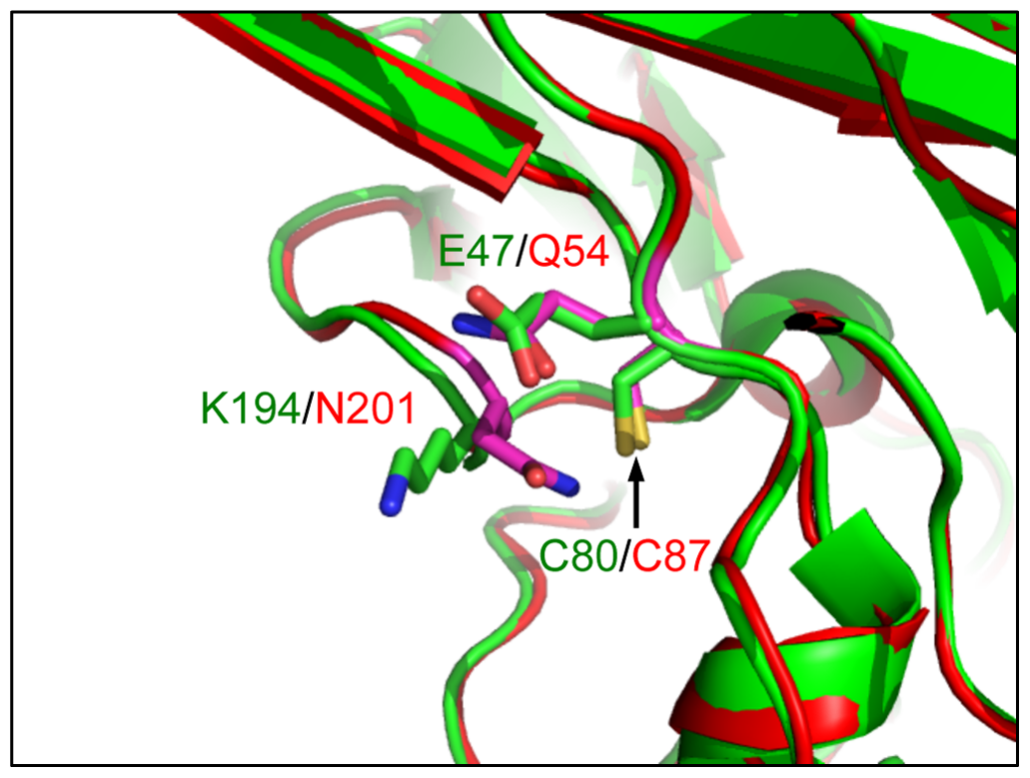
Structural difference around C80 between JCV Vp1 and SV40 Vp1. Vp1 structures of JCV and SV40 are shown in green and red, respectively. The different residues within 4 Å of C80 of the Vp1s are shown as stick models. An arrow indicates C80 in JCV Vp1 and C87 in SV40 Vp1.

The two non-VLP-forming mutant Vp1s, C80A and C247A, were synthesized normally in HeLa cells but were degraded over 12 h, leading to low steady-state levels. In regard to the other C80 substitution mutants expressed in HeLa cells, the C80S mutant Vp1 was just as unstable as C80A. Conversely the C80T mutant Vp1 was stable, which is analogous to the fact that a threonine is naturally present at the corresponding position in MPyV Vp1. For the C80A mutant Vp1, a local structural perturbation brought about by a side-chain alteration at the cysteine residue could have caused the protein to be degradation-prone protein in HeLa cells. C87 of SV40 Vp1 is analogous to C80 of JCV Vp1. C87A mutant SV40 preserves viral viability [12]. A comparison of the structure of JCV Vp1 and SV40 Vp1reveals 2 differences within 4 Å of C80: E47 in JCV Vp1 and Q54 in SV40 Vp1, and K194 in JCV Vp1 and N201 in SV40 Vp1 (Fig. 8). The differences in the local structures might explain the stability difference between C80A mutant JCV Vp1 and C87A mutant SV40 Vp1. In regard to JCV C247, almost any substitutions at this cysteine residue that we made with well-conserved structural elements produced unstable mutant proteins (Fig. 4E). C247 lies on the G2H loop, which is absolutely conserved among polyomavirus [12]. Perhaps this lack of stability seen in all JCV C247 substitution mutants is analogous to that reported for certain SV40 C254 mutants, for which viability is affected by a local structural perturbation of Vp1's G2H loop due to substitution of the cysteine with long side chain amino acids [12]. We thus conclude that the low steady-state levels of the JCV mutant Vp1s resulted from two types of structural disturbances: one in the local structure of monomer surrounding C247, and the other at the interface of pentamer-pentamer contact sites.

When the cysteine point mutations were introduced into the JCV genome and the mutant genomes were transfected into SVG-A cells, we found that the infectivities of C80A and C247A mutants, which produced unstable mutant Vp1s in HeLa cells, were significantly lower than those of the WT and of the other cysteine mutants. The decreased infectivity of the two mutants came about for different reasons: the C80A Vp1 was not capable of nuclear localization, whereas the C247A Vp1 localized in the nucleus. It is noteworthy that we observed two distinct mutant phenotypes arising from single cysteine mutations in JCV Vp1. One phenotype, exhibited by the C80 mutant of JCV Vp1, relates to what occurs in the cytoplasm; while the other phenotype, exhibited by the C247 mutant, relates to what occurs in the nucleus. Polyomavirus Vp1 is traditionally observed as pentamers but not monomers. C49A–C87A pair mutant SV40 Vp1, which is unable to make Vp1 folding and to form pentamer, is located in cytoplasm [13]. Our results revealed that discrete steps in the formation of infectious JCV virions are defined by two JCV Vp1 cysteines: C80 functions in Vp1 folding in the cytoplasm and C247 functions at the nuclear stage of virion assembly.

Our application of an *in vitro* translation system with isolated protein components, complete with disulfide-linking agents, has led to the production of stable mutant Vp1s, including C80A and C247A Vp1s, that are unstable in HeLa cells. Given the formation of stable mutant Vp1s *in vitro*, we observed that the two single cysteine-to-alanine substitution mutant Vp1s exhibited strikingly different biochemical properties: the C247A Vp1 could form pentamers, whereas the C80A Vp1 could not oligomerize. Our results suggest distinct roles for these two cysteines during Vp1 folding and oligomerization. Although no disulfide bonds are present in the Vp1 pentamers of the mature SV40 particle [8], [9], transient disulfide bonds form during the folding and pentamer formation processes of SV40 Vp1 [10]. The JCV Vp1 pentamer structure reveals an absence of disulfide bonds in the Vp1 pentamers, and C80 is buried in the hydrophobic core of Vp1 (Fig. 1A and B) [5]. C80T Vp1 forms pentamers (Fig. 6C). Therefore, a local structure of C80, rather than disulfide bond formation, is essential for Vp1 pentamer formation.

The stability of all mutant Vp1s produced in the cell-free protein synthesis system contrasts with the instability of the degradation-prone C80A and C247A mutant Vp1s made in HeLa cells. Protein degradation was not dependent on the known ubiquitin-proteasome system or autophagy. We thus suggest that HeLa cells must contain cellular factors that recognize certain structure-based abnormalities, and that such proteins must have recognized C80A and C247A mutant Vp1s as unfit in forging folding and assembly, hence promoting the mutant Vp1s' degradation and instability. Our observation parallels a finding reported in prokaryotes: mutant α-subunit proteins of *Bacillus* PS3 that are unstable in the host and are degraded by some cellular protein degradation factor are stable when synthesized using an *in vitro* system [29]. What these cellular factors are remains to be determined.

In the present study, we identified two cysteine residues, C80 and C247, in JCV Vp1 as two determinants of the JCV capsid formation. The results contribute to the understanding of the mechanisms of virion formation in polyomaviruses.

## Supporting Information

Figure S1 **Subcellular localization of mutant Vp1s.** SVG-A cells transfected with JCV genomes encoding WT or mutant Vp1s for 3 days were subjected to immunofluorescence analysis for the Vp1s' subcellular localization (*green*). Cell nuclei were counterstained with DAPI (*blue*). Merged images of DAPI and Vp1 are also represented.
(TIF)
