# Supplementary figures and images for: Cysteine Residues in the Major Capsid Protein, Vp1, of the JC Virus Are Important for Protein Stability and Oligomer Formation
Source: PLoS One. 2013 Oct 9;8(10):e76668. doi: 10.1371/journal.pone.0076668 (PMC3793911; doi:10.1371/journal.pone.0076668)

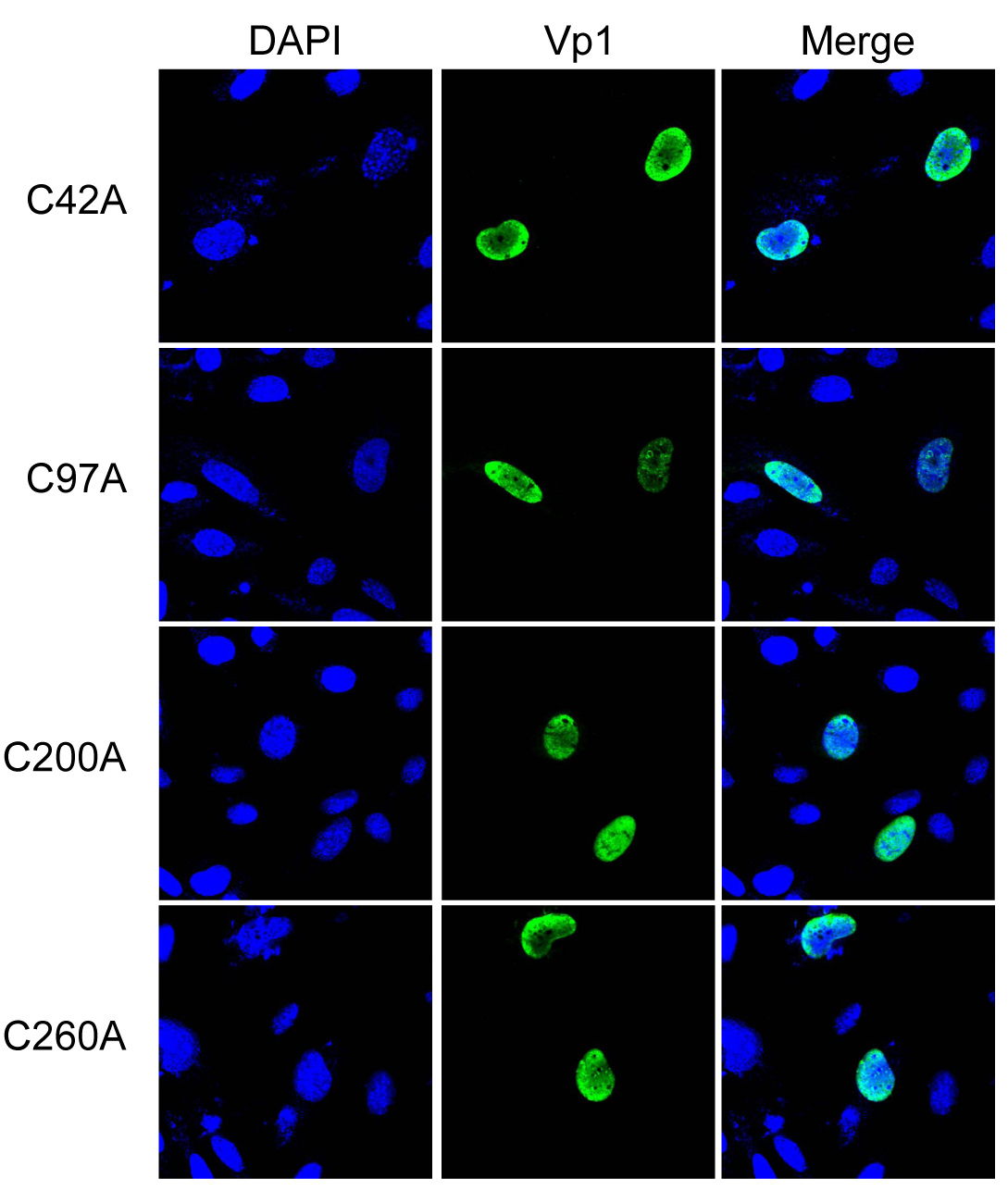

Supplement: Figure S1 — Subcellular localization of mutant Vp1s. SVG-A cells transfected with JCV genomes encoding WT or mutant Vp1s for 3 days were subjected to immunofluorescence analysis for the Vp1s' subcellular localization (green). Cell nuclei were counterstained with DAPI (blue). Merged images of DAPI and Vp1 are also represented. (TIF) [file pone.0076668.s001.tif]
